# Standardized artificially created stable pertrochanteric femur fractures present more homogenous results compared to osteotomies for orthopaedic implant testing

**DOI:** 10.1186/s12891-021-04234-4

**Published:** 2021-04-20

**Authors:** J.F. Schader, I. Zderic, D. Gehweiler, J. Dauwe, K. Mys, C. Danker, Y. P. Acklin, C. Sommer, B. Gueorguiev, K. Stoffel

**Affiliations:** 1 AO Research Institute Davos, Clavadelerstrasse 8, 7270 Davos Platz, Switzerland; 2 University of Basel, Basel, Switzerland; 3 Department of Surgery, Cantonal Hospital Graubuenden, Chur, Switzerland; 4 Department of Trauma Surgery, UZ Leuven, Leuven, Belgium; 5 University Hospital Basel, Basel, Switzerland

**Keywords:** Fracture model, Fracture standardization, Osteotomy, Stable pertrochanteric fracture, Fracture line analysis, Proximal femur fracture, Fracture biomechanics

## Abstract

**Background:**

With regard to biomechanical testing of orthopaedic implants, there is no consensus on whether artificial creation of standardized bone fractures or their simulation by means of osteotomies result in more realistic outcomes. Therefore, the aim of this study was to artificially create and analyze in an appropriate setting the biomechanical behavior of standardized stable pertrochanteric fractures versus their simulation via osteotomizing.

**Methods:**

Eight pairs of fresh-frozen human cadaveric femora aged 72.7 ± 14.9 years (range 48–89 years) were assigned in paired fashion to two study groups. In Group 1, stable pertrochanteric fractures AO/OTA 31-A1 were artificially created via constant force application on the anterior cortex of the femur through a blunt guillotine blade. The same fracture type was simulated in Group 2 by means of osteotomies. All femora were implanted with a dynamic hip screw and biomechanically tested in 20° adduction under progressively increasing physiologic cyclic axial loading at 2 Hz, starting at 500 N and increasing at a rate of 0.1 N/cycle. Femoral head fragment movements with respect to the shaft were monitored by means of optical motion tracking.

**Results:**

Cycles/failure load at 15° varus deformation, 10 mm leg shortening and 15° femoral head rotation around neck axis were 11324 ± 848/1632.4 ± 584.8 N, 11052 ± 1573/1605.2 ± 657.3 N and 11849 ± 1120/1684.9 ± 612.0 N in Group 1, and 10971 ± 2019/1597.1 ± 701.9 N, 10681 ± 1868/1568.1 ± 686.8 N and 10017 ± 4081/1501.7 ± 908.1 N in Group 2, respectively, with no significant differences between the two groups, *p* ≥ 0.233.

**Conclusion:**

From a biomechanical perspective, by resulting in more consistent outcomes under dynamic loading, standardized artificial stable pertrochanteric femur fracture creation may be more suitable for orthopaedic implant testing compared to osteotomizing the bone.

## Introduction

Medical devices have to undergo a long and strictly regulated evaluation before approval for release on the market [1]. Biomechanical testing is crucial for development of orthopaedic implants with regard to both premarket evaluation and post market vigilance [2]. Usually, biomechanical behavior of orthopaedic implants in fractured bones is investigated via fracture simulation by means of osteotomies [3]. This ensures a standardized preparation of the specimens [4]. However, the biomechanical behavior of osteotomized bones, e.g. their low stability under shear stress in the osteotomy plane, might differ when compared to real fractures seen in daily traumatological practice – the situation the orthopaedic implants are designed for. Therefore, it is important to know whether specimens with bone fractures, created by controlled breaking in laboratory conditions, would be more suitable for biomechanical testing of orthopaedic implants than osteotomized specimens. So far, this problem has been partially addressed by creation of e.g. rabbit tibia shaft fractures to investigate bone healing following different fracture fixation concepts [5]. However, the biomechanical properties of fractured bones might not only play a crucial role in vivo, but also when testing fresh-frozen human cadaveric specimens. Fracture creation using a drop tower is often applied to understand the pathomechanics of specific fracture patterns [6]. However, it often results in complex multi-fragmentary patterns that cannot be reproducibly standardized. Simulating a sideways fall on the greater trochanter is another common concept to investigate proximal femur fractures, but it also can uncontrollably result in all possible kinds of fracture types ranging from cervical over intertrochanteric to subtrochanteric fractures [7]. Investigation of fracture patterns and specifically their prediction is subject of current research [8]. Nevertheless, researchers often disagree on how close one needs to reflect the clinical reality of fractures at the expense of their standardization. From an engineering point of view, the main question is how to create a bone fracture in a standardized way, while still obeying the bone architecture without interference with the natural progression of the fracture formation. On the other hand, from a biomedical point of view the question is whether there is a significant difference between the behavior of fractured and osteotomized human cadaveric bones used for orthopaedic implant testing. Therefore, the aims of this study were (1) to investigate the possibility for creation of standardized pertrochanteric bone fractures, and (2) to compare their biomechanical stability versus osteotomized bones for orthopaedic implant testing.

## Materials and methods

### Specimens preparation

Eight pairs of fresh-frozen proximal human cadaveric femora from one female and seven male donors aged 72.7 ± 14.9 years (mean ± standard deviation, SD; range 48–89 years) were used in this study. The specimens were collected from an accredited donation program (Science Care, Inc., Phoenix, AZ, USA). All donors gave their informed consent inherent within the donation of the anatomical gift statement during their lifetime. The specimens underwent computed tomography (CT) scanning (Revolution EVO, GE Medical Systems AG, Switzerland) at a slice thickness of 0.63 mm to ensure no evidence of any pathology and to measure their bone mineral density (BMD) within a cylinder of 20 mm diameter and 30 mm length, located in the center of the femoral head, with the use of a calibration phantom (European Forearm Phantom QRM-BDC/6, QRM GmbH, Möhrendorf, Germany). Subsequently, the femora were assigned in paired fashion to two study groups.

In Group 1, stable pertrochanteric fractures AO/OTA 31-A1 were created in each right femur. By definition, a pertrochanteric fracture line is located in the region between the greater and lesser trochanter [9]. A stable fracture A1 is defined by a lateral wall thickness of more than 20.5 mm as measured from a reference point located 3 cm below the innominate tubercle of the greater trochanter to the intersection with the fracture line in an angle of 135° from this point on the anteroposterior x-ray image [9]. Although several previous studies [10, 11] reported a high interobserver variability in defining the subgroups A1.1-A1.3 of this fracture type (A1), this definition corresponds to all of them.

**Fig. 1:**
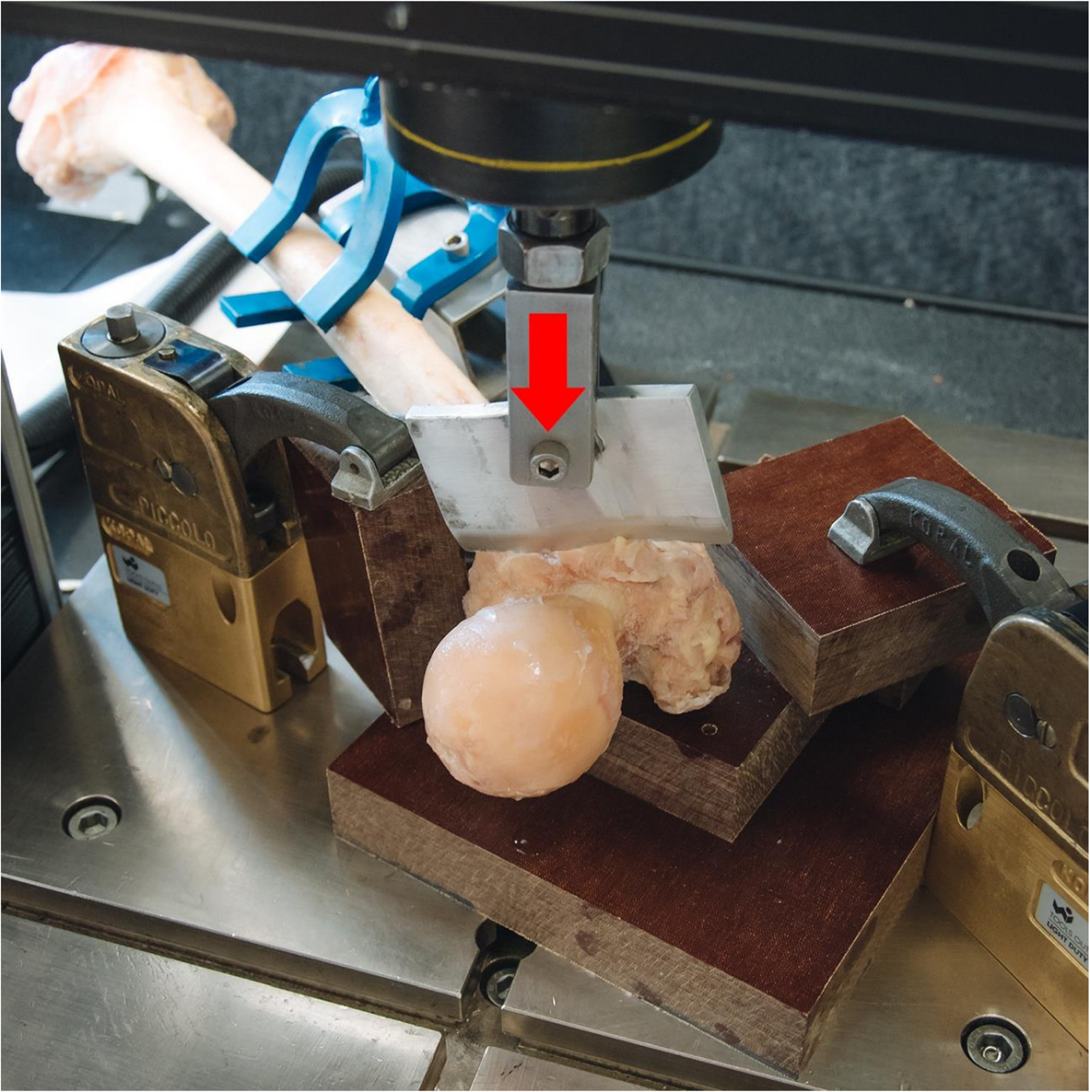
Setup with a specimen mounted for creation of a stable pertrochanteric fracture by means of a blunt guillotine blade, with vertical arrow indicating the force direction

**Fig. 2:**
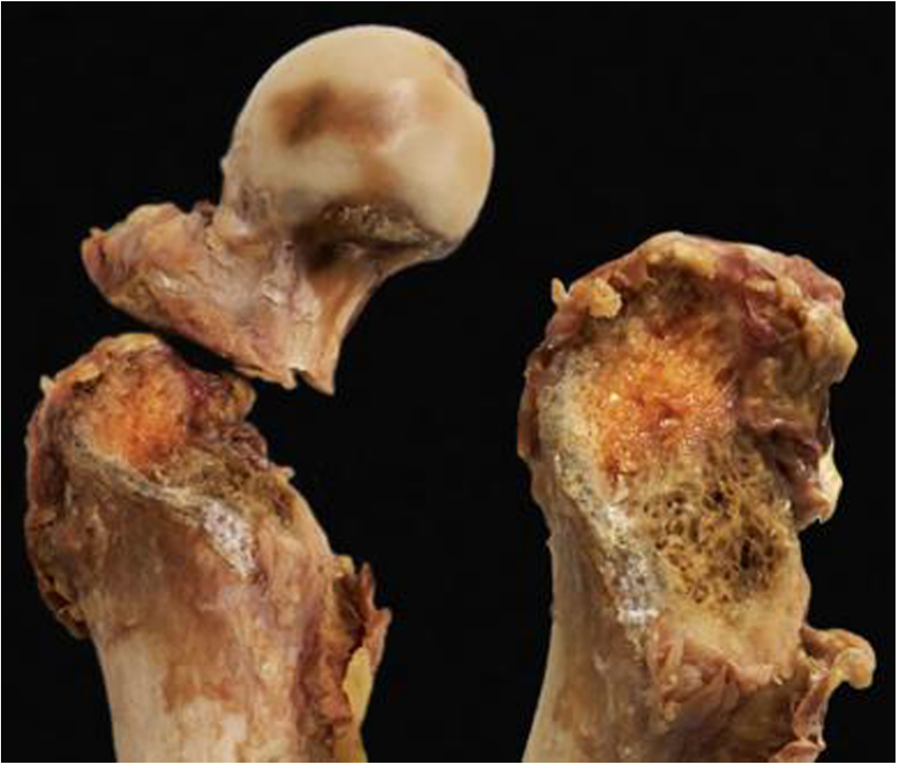
Visualization of the created patterns of a pertrochanteric fracture AO/OTA 31-A1 in Group 1

All fractures were created with a custom-made blunt guillotine blade, which was connected to the actuator of a material testing machine (#5866, Instron, Norwood, MA, USA) equipped with a 10 kN load cell. The blade was positioned on the anterior cortex of the bone in an angle of 41° to the femoral shaft with the cutting direction being adjusted to the individual anteversion angle in the frontal plane by supporting the bone on three fixed points located at the lesser trochanter, the greater trochanter, and the distal condyle plane (Fig. 1). The determination of the fracture angle was based on findings from a previous radiologic study analyzing 164 anteroposterior radiographs of the hip and pelvis from patients with trochanteric fractures [12]. An angle of 41° ± 8° with respect to the femoral shaft axis was reported for the cases with two-part fractures among those patients. In order to create a stable fracture, the blade location was specified as starting from the innominate tubercle of the greater trochanter and ending in the region proximal to the lesser trochanter. A constant actuator displacement rate of 5 mm/s was applied up to a depth of 10 mm, which proved to be enough in conducted pilot tests to penetrate the first (anterior) cortex in the pertrochanteric region and initiate the artificial creation of standardized fracture type AO/OTA 31-A1. Consequently, the fracture line of each bone proceeded its natural propagation according to the trabecular architecture of the femur and resulted in a full AO/OTA 31-A1 fracture type pattern (Fig. 2).

In Group 2, an osteotomy, simulating stable femoral pertrochanteric fracture type AO/OTA 31-A1, was created in each left femur with an oscillating saw blade of 0.8 mm thickness. For that purpose, a custom-made cutting guide was designed and aligned with the lateral cortex of the femoral shaft. Starting from the innominate tubercle of the greater trochanter, the osteotomy was set in an angle of 41° to the shaft axis in the frontal plane and an angle of 15° in the sagittal plane, the latter chosen to consider the anteversion of the femoral neck.

**Fig. 3:**
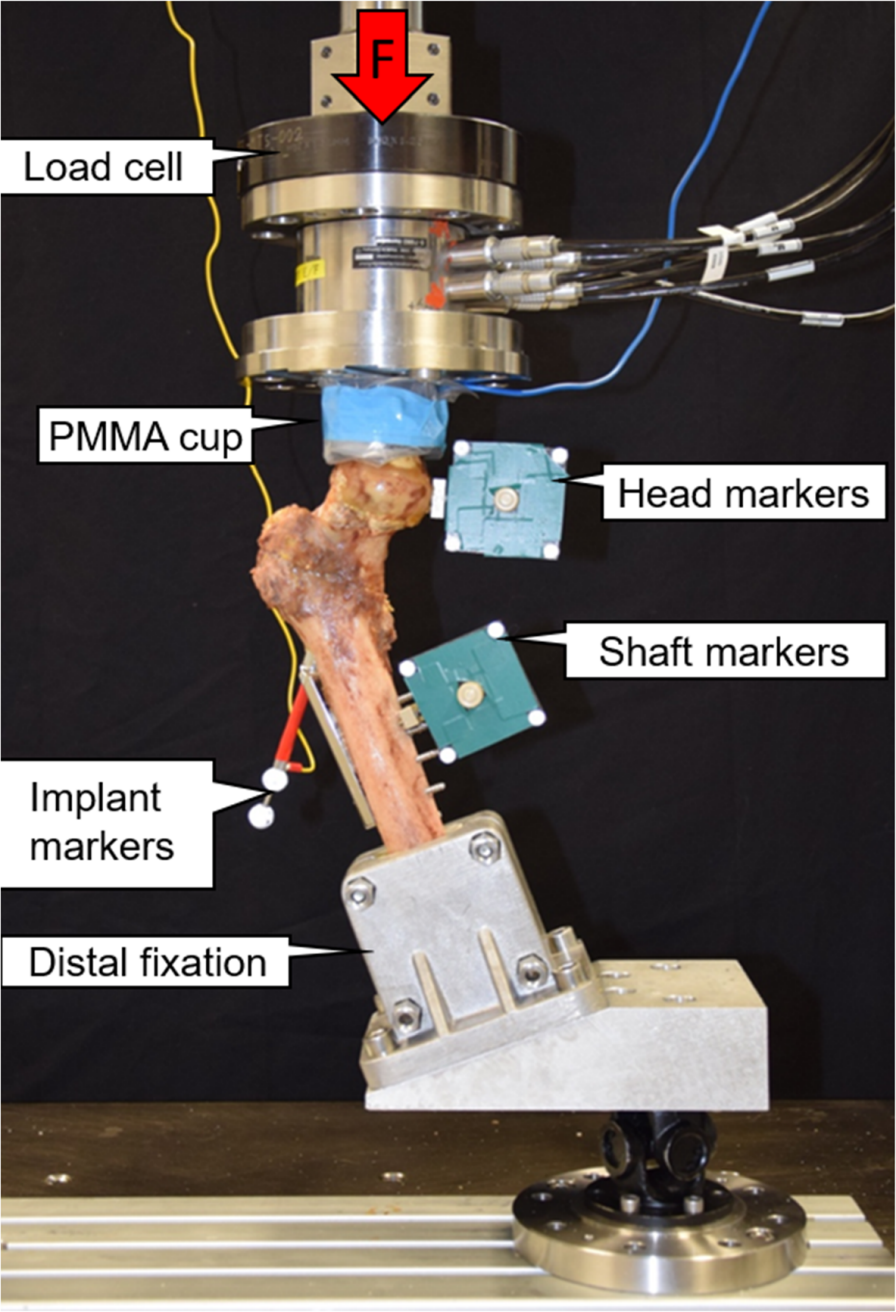
Test setup with a specimen mounted for biomechanical testing, with vertical arrow indicating the loading direction

All specimens were anatomically reduced and subsequently implanted with a Dynamic Hip Screw (DHS, DePuy Synthes, Zuchwil, Switzerland) according to the manufacturer’s guidelines. The lag screw was positioned in center-center position and a tip-apex distance of less than 25 mm was considered to minimize the risk of cut-out failure. No additional antirotation screw was used. All femora were cut distally at a length of 250 mm, measured from the tip of the greater trochanter, and the distal 65 mm were embedded in polymethylmethacrylate (PMMA, SCS-Beracryl D-28, Suter Kunststoffe AG, Fraubrunnen, Switzerland) in preparation for biomechanical testing. Finally, retro-reflective marker sets were attached to the shaft, femoral head fragment and DHS head element for optical motion tracking (Fig. 3).

### Creation of a mean shape model

All sixteen specimens were scanned in both intact and fractured/osteotomized states using a clinical CT scanner (Revolution EVO, GE Medical Systems AG, Switzerland) at a slice thickness of 0.63 mm. Cortex masks of the different fragments were generated on the fractured/osteotomized scans in a semi-automatic procedure using in-house developed script in Matlab (R2019a, MathWorks, Natick, MA, USA) and C + + code [13]. These scans were then registered on the intact scans in order to locate the fracture lines using Amira software package (Amira 6.2, FEI Company, Hillsboro, Oregon, USA). Based on the scans of intact bones, a mean shape model was created by manual positioning of the anatomical and segmental landmarks as previously described [14]. The left bone sides were mirrored to create one mean shape model for both right and left femora. The fracture lines were automatically created using custom made Amira scripts and Visualization Toolkit (VTK, Kitware Inc., Clifton Park, NY, USA). The closest points of the registered fracture parts were automatically determined and interpolated to a single fracture line. Each individual fracture/osteotomy line was homologized and transferred to the mean shape model. One average line and two standard-deviation lines were calculated for all eight individual lines of each group and projected onto the surface of the mean shape model. The standard deviation values corresponding to each separate point on the average fracture/osteotomy line – calculated via the distances between the respective eight individual points and their projections on the average fracture/osteotomy plane – were considered for statistical equality/divergence comparison analysis of the two different procedures for creation of the stable pertrochanteric fractures by means of either constant force application (Group 1) or osteotomizing (Group 2).

### Biomechanical testing

Biomechanical testing was performed on a servohydraulic test system (Bionix 858, MTS Systems, Eden Prairie, MN, USA) equipped with a 4 kN load cell at room temperature (20 °C) in dry environment. Both test setup and loading protocol were adopted from previous studies [15–17] (Fig. 3). The specimens were tested in 20° lateral angulation (adduction) of the femoral shaft [15]. Load transfer between the load cell – attached to the machine actuator – and femoral head was ensured via an interconnected PMMA shell, simulating the acetabulum. A special custom-made foil from electro-conducting material was implemented at the corresponding articular surface of the cup and used for immediate automatic detection of implant cutting through the femoral head (cut-out) during testing and interruption of the test procedure to prevent implant damage as soon as an electric contact occurred. The cranial area for proximal load transfer to the bone was localized at the superior aspect of the femoral head. Distally, the specimen was attached to the machine frame via a cardan joint.

Each specimen was loaded in compression along the machine axis, starting with a quasi-static ramp from 50 to 200 N at a rate of 15 N/sec, followed by progressively increasing cyclic loading at 2 Hz with physiologic profile of each cycle [15, 17]. Keeping the valley load of each cycle at 200 N, its peak load started at 500 N and then increased at a rate of 0.1 N/cycle until failure of the bone-implant construct. The application of progressively increasing cyclic loading allows to achieve construct failure of specimens with different bone quality within a predefined number of cycles and has been demonstrated as useful in previous studies [17, 18]. The test stop criteria considered either cut-out of the implant through the femoral head, 30 mm vertical displacement of the machine actuator relative to the test beginning, or reaching an axial load of 4 kN.

### Data acquisition and evaluation

Machine data in terms of axial displacement (mm) and axial load (N) were recorded from the machine controllers at 128 Hz. Based on this, initial axial construct stiffness was calculated from the ascending slope of the load-displacement curve from the initial quasi-static ramp within the linear range between 100 and 200 N.

Three-dimensional coordinates of the retro-reflective markers attached to the bone and implant were collected at 100 Hz using 5 infrared cameras (ProReflex MCU 120, Qualisys AB, Gothenburg, Sweden) to investigate the femoral head movements with respect to the shaft and implant in all six degrees of freedom. Based on the motion tracking data, varus deformation was defined as the relative femoral head-to-shaft rotational movement in the coronal plane. Furthermore, leg shortening was derived from the movement of the head center along the shaft axis. Finally, the rotation of the femoral head around the neck axis was evaluated. For that purpose, the neck axis was reconstructed via virtual rotation of the shaft coordinate system around its anteroposterior axis by the amount given from the measured caput-collum-diaphyseal (CCD) angle of each femur. The outcome values of these parameters were analyzed after 1000, 5000 and 10,000 cycles in peak loading conditions to evaluate the degradation of the construct stability over the course of cycles.

Furthermore, 15° varus deformation, 10 mm leg shortening and 15° femoral head rotation around the neck axis – considered with respect to the beginning of the cyclic test – were defined as clinically relevant failure criteria, and the numbers of cycles until fulfilment of each of these criteria in peak loading condition were calculated for each specimen separately.

Anteroposterior radiographic images were taken at the beginning (50 N) and the end (200 N) of the quasi-static ramp, and then at timed intervals every 250 cycles during the cyclic test at valley loading (200 N) using a triggered C-arm (Siemens ARCADIS Varic, Siemens Medical Solutions AG, Erlangen, Germany). X-ray images taken at the end of each test served to evaluate the catastrophic failure modes of the specimens.

Statistical analysis was performed with SPSS software package (IBM SPSS Statistics, V23, IBM, Armonk, NY, USA). Shapiro-Wilk test was conducted to screen and prove the normality of data distribution. Independent-Samples t-test was applied to detect significant differences during the equality/divergence analysis for comparison of the two different procedures for creation of the stable pertrochanteric fractures in the two groups. Significant differences between the groups regarding BMD, axial stiffness and cycles to 15° varus deformation, 10 mm leg shortening and 15° rotation of the femoral head around the neck axis were identified with Paired-Samples t-tests. General Linear Model Repeated Measures test was applied to detect significant differences between the groups with regard to the parameters evaluated over the three time points after 1000, 5000 and 10,000 cycles. The homogeneity of biomechanical fracture stability was compared between the two groups by conducting a Wilcoxon-Signed Rank test over the pooled SDs of the outcomes derived from the three motion tracking parameters of interest investigated after 5000 test cycles, and the cycles to clinically relevant failure. The latter non-parametric paired approach was applied only to relate the corresponding ranks within the pooled SDs of the different parameters, because such an approach is anticipated to be sound when comparing values emerging from different physical variables. Level of significance was set to 0.05 for all statistical tests.

## Results

### Mean shape model

**Fig. 4:**
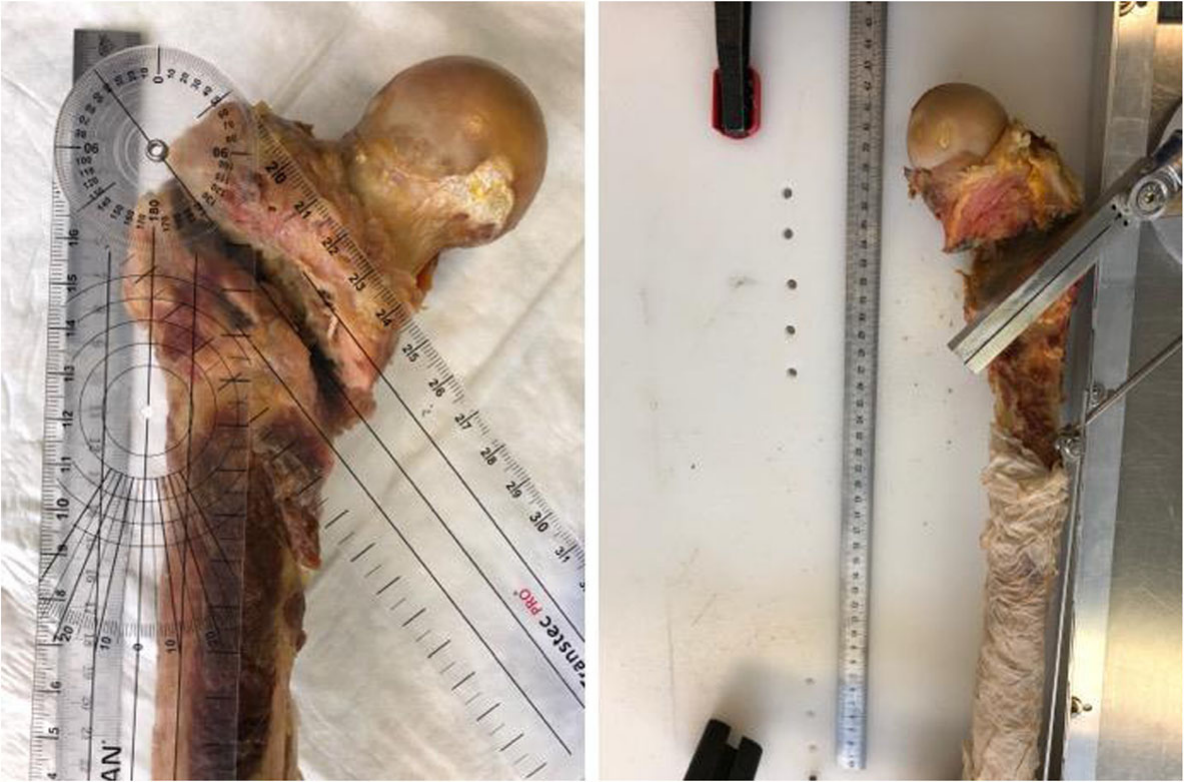
Exemplified images of a fractured (left) and an osteotomized (right) specimen; on the left, fracture angle of 41° degree is displayed as indicated with a goniometer; on the right, osteotomy saw guide placed in an angle of 41° is presented

**Fig. 5:**
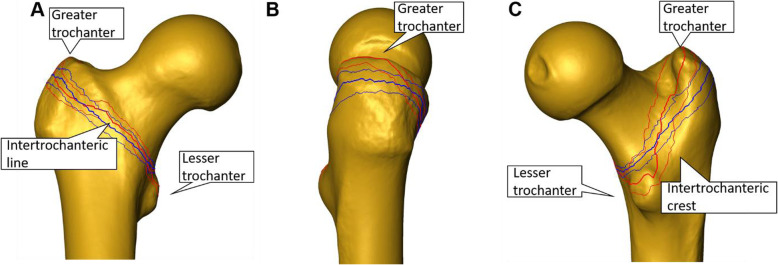
**a**-**c** Visualization of the mean shape model and both its mean fracture line (thick red) with SD (thin red) and mean osteotomy line (thick blue) with SDs (thin blue) in **a** anteroposterior, **b** lateral, and **c** posteroanterior view

Exemplified images of a fractured and an osteotomized femur are presented in Fig. 4. The created mean shape model, both its mean fracture and osteotomy lines, and the corresponding standard-deviation lines are visualized in Fig. 5. Both mean fracture and osteotomy lines were located within the pertrochanteric area in anteroposterior, lateral and posteroanterior view. The standard deviation values of the average fracture (Group1) and osteotomy (Group 2) lines were 6.03 ± 0.81 mm and 4.25 ± 1.33 mm, respectively, and differed significantly from each other, *p* < 0.001. In anteroposterior view, there was a predominant overlapping of the fracture and the osteotomy lines along the intertrochanteric line. In lateral and posteroanterior view, however, these two lines differed in their progression within the bone. Whereas the osteotomy lines were with the same inclination towards the long bone axis as in anterior view, the fracture lines coincided with the intertrochanteric crest in posterior view and therefore were more vertical to the shaft axis than the osteotomy lines.

**Fig. 6:**
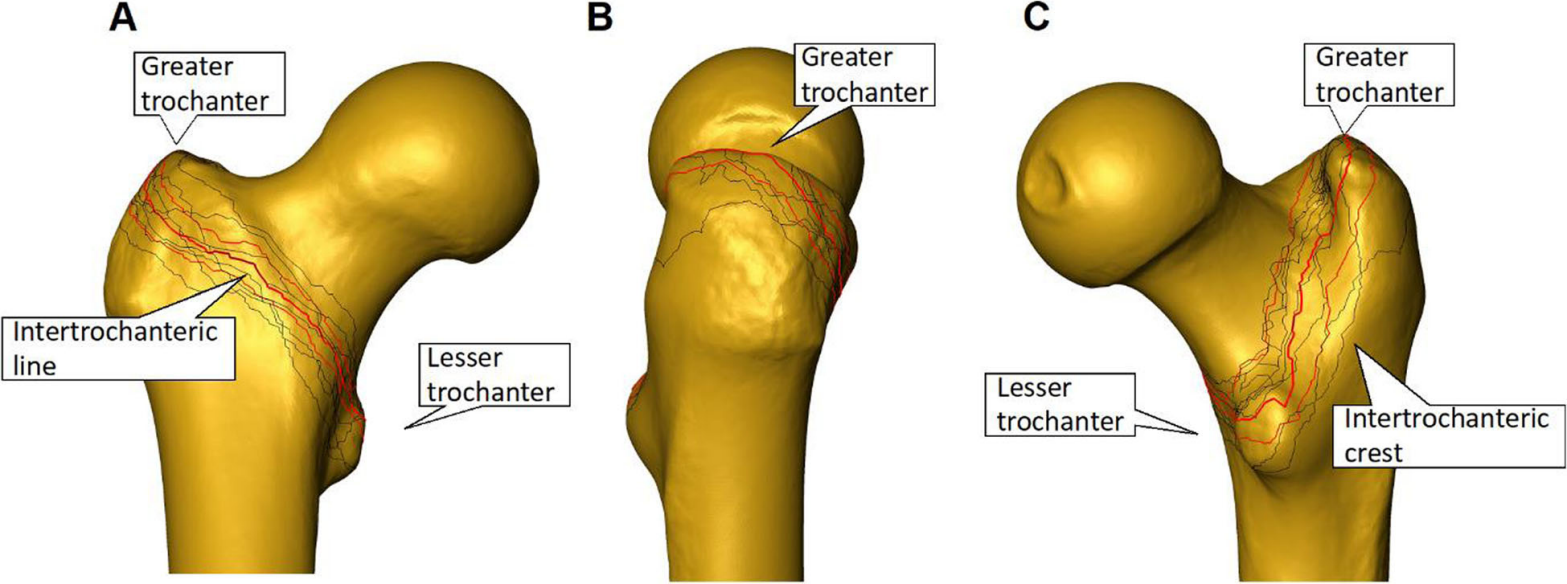
**a-c **Visualization of the mean shape model and the fracture lines of all eight fractured specimens (black) together with their mean (thick red) and SD (thin red) lines in **a** anteroposterior, **b** lateral, and **c** posteroanterior view

Figure 6 presents the fracture lines of all eight fractured specimens with their mean and SDs lines as projected on the mean shape model. All fracture lines were located within the pertrochanteric area reaching to the basicervical line of the femoral neck. According to the definition for stable pertrochanteric fractures (AO/OTA 31-A1) described above, lateral wall thickness was more than 20.5 mm. No fracture lines were found distally to the lesser trochanter, and despite the involvement of the latter in three specimens, the posterior medial wall always remained intact.

### Biomechanical testing

#### Morphometrics

The CCD angles were comparable between fractured (131.5 ± 4.9°) and osteotomized (132.1 ± 4.4°) femora, *p* = 0.603. BMD values were 294.6 ± 44.8 mgHA/cm^3^ in Group 1 and 298.6 ± 50.8 mgHA/cm^3^ in Group 2, *p* = 0.621.

#### Axial stiffness

Axial stiffness was 137.9 ± 42.3 N/mm for fractured and 114.5 ± 30.2 N/mm for osteotomized bones, with no significant difference between them, *p* = 0.241.

#### Movements at the fracture site

Descriptive outcome measures of the predefined fracture displacement parameters – varus deformation, leg shortening and femoral head rotation around neck axis – evaluated over the three time points after 1000, 5000 and 10,000 cycles are summarized in Table 1, showing significant increase of each parameter between those cycle numbers, *p* ≤ 0.011. However, the differences between the groups remained non-significant for each parameter, *p* ≥ 0.093.

**Table 1:** Outcome measures of the femoral head movements with respect to the shaft in the two study groups with fractured and osteotomized specimens after 1000, 5000 and 10,000 cycles, in terms of mean and standard deviation, together with *p*-values from the statistical comparisons between the groups and over cycles

Group	1000 cycles	5000 cycles	10,000 cycles	*p*-value (difference between groups)
*Varus deformation [°]*				
* Fractured*	0.5 ± 0.2	2.4 ± 0.6	8.5 ± 2.7	*p* = 0.501
* Osteotomized*	0.7 ± 0.5	3.0 ± 2.5	10.7 ± 8.3	
* p-value over cycles*	*p* < 0.001			
*Leg shortening [mm]*				
* Fractured*	0.8 ± 0.3	2.5 ± 1.6	5.8 ± 4.5	*p* = 0.424
* Osteotomized*	0.8 ± 0.5	2.6 ± 2.0	15.9 ± 12.4	
* p-value over cycles*	*p* = 0.001			
*Rotation around neck axis [°]*				
* Fractured*	0.4 ± 0.4	1.5 ± 1.4	4.8 ± 3.1	*p* = 0.093
* Osteotomized*	1.9 ± 2.4	5.4 ± 8.3	13.4 ± 15.2	
* p-value over cycles*	*p* = 0.011			

#### Cycles to failure and failure loads

**Fig. 7:**
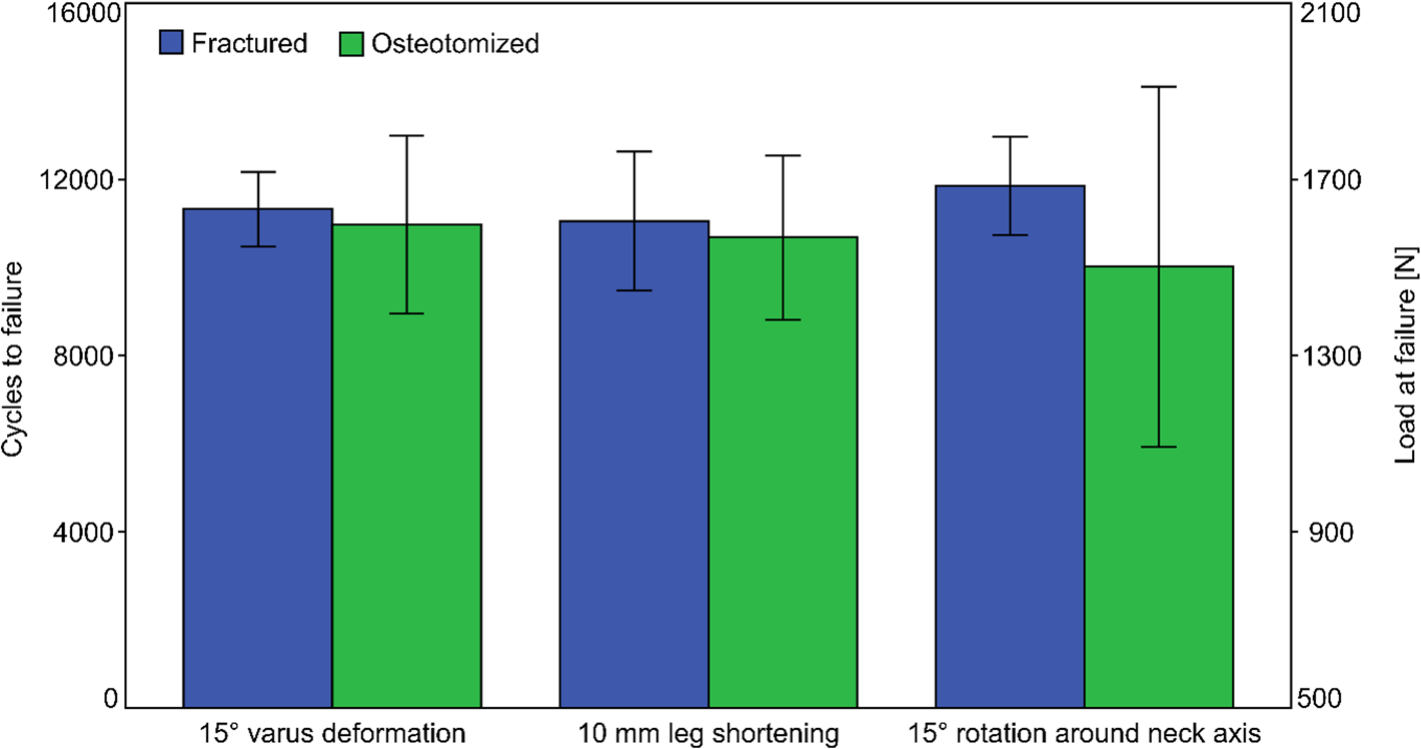
Diagram presenting cycles to failure and failure load in the two study groups with fractured and osteotomized specimens according to the clinically relevant criteria 15° varus deformation, 10 mm leg shortening and 15° femoral head rotation around the neck axis

Cycles to 15° varus deformation, 10 mm leg shortening and 15° femoral head rotation around neck axis were 11,324 ± 848, 11,052 ± 1573 and 11,849 ± 1120 in Group 1, and 10,971 ± 2019, 10,681 ± 1868 and 10,017 ± 4081 in Group 2, respectively, with no significant differences between them for each separate outcome, *p* ≥ 0.233 (Fig. 7). The corresponding peak failure loads at 15° varus deformation, 10 mm leg shortening and 15° femoral head rotation around neck axis were 1632.4 ± 584.8 N, 1605.2 ± 657.3 N and 1684.9 ± 612.0 N in Group 1, and 1597.1 ± 701.9 N, 1568.1 ± 686.8 N and 1501.7 ± 908.1 N in Group 2, respectively, with no significant differences between them for each separate outcome, *p* ≥ 0.233.

#### Homogeneity of outcomes

Pooled SDs among the outcome measures axial stiffness and femoral head movements (varus deformation, leg shortening and femoral head rotation around neck axis) with respect to the shaft after 1000, 5000 and 10,000 cycles, as well as cycles to 15° varus deformation, 10 mm leg shortening and 15° femoral head rotation around neck axis, were significantly smaller in Group 1 than in Group 2, *p* = 0.028.

#### Modes of catastrophic failure

**Fig. 8:**
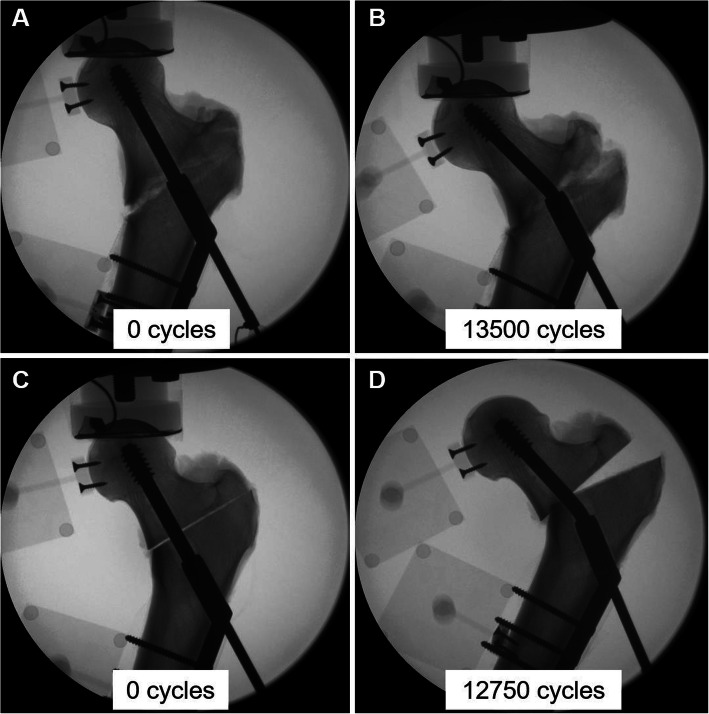
Exemplified x-rays of specimens from Group 1 (fractured: **a**, **b**) and Group 2 (osteotomized: **c**, **d**) – both fixed with DHS – at initial (**a**, **c**) and final (**b**, **d**) stage of testing, with indicated corresponding number of cycles at test begin and cycles to 10 mm leg shortening

All specimens failed in a combination of varus deformation, leg shortening and implant bending at the region where the lag screw extended from the barrel (Fig. 8). No implant or shaft screw breakage, or femoral shaft fracture occurred.

## Discussion

This is the first study describing artificial creation of more realistic standardizable stable pertrochanteric proximal femur fractures for biomechanical cadaveric orthopaedic implant testing purposes. The achieved fracture locations and paths were in good agreement with the aimed ones.

It is known that pertrochanteric proximal femur fractures are common in daily surgical practice and can be fixed with a variety of different available implants [19]. Therefore, the introduction of this novel approach for standardized fracture creation was focused on such fracture type patterns. Another reason for this choice were the clearly identifiable bony landmarks, such as the greater and lesser trochanter, the intertrochanteric crest, the transition to the femoral neck and femoral shaft, as well as the angle of anteversion. Our innovative approach proved that it is feasible to artificially create standardized frequently occurring fractures within a complex anatomic region.

The decision to fix a stable pertrochanteric fracture with an extramedullary device was based on the fact that the test setup should be as simple as possible to minimize confounding and biasing factors, such as e.g. fracture reduction or implant performance. Although stable pertrochanteric fractures can be treated with extra- or intramedullary devices, we expected higher shear forces along the fracture/osteotomy surface with such an extramedullary device as the DHS and therefore more distinguished differences in the outcomes when comparing these two techniques for fracture creation.

Various techniques were developed to simulate closed fractures with intact soft tissue for investigation of bone healing [20, 21] or teaching purposes. While simple transverse shaft fractures of the tibia or femur were mainly created with a drop tower in the aforementioned studies, the use of their approach in the proximal femur region would most likely result in comminuted fractures due to the different and complex bone geometry of the latter [22]. Therefore, and in contrast to these studies, we utilized a blade force generated at a constant velocity to fracture the bone and achieve high reproducibility of the fracture creation. Without creating a predetermined initial breaking aspect by means of the guillotine blade, applying force to the intact bone would probably have resulted in a variety of fracture types, predominantly femoral neck fractures, as experienced in other studies [7, 8]. The economic costs of this approach are relatively low if a standard hydraulic material testing machine equipped with a 10 kN load cell is available. The metal blade was formed manually with a bench vice. All eight fractures were created within one day by two surgeons. Investigation of fracture patterns plays a major role in the understanding of fracture pathomechanics and might help avoid intraoperative pitfalls [23]. Fracture characteristics might also depend on the bone quality [24].

Significant difference was demonstrated between the two procedures for creation of pertrochanteric fractures by means of fracturing or osteotomizing. In contrast to the artificially set plane osteotomies in Group 2, the overall fracture patterns in Group 1 followed anatomical landmarks (Fig. 4). Therefore, the artificially created fractures seem to resemble native fractures better than osteotomies regarding location and morphology.

Three clinically relevant failure criteria were chosen to depict as close as possible the clinical reality of biomechanical fracture performance. Due to the lateral location of the greater trochanter relative to the center of rotation, the actions of the attached pelvitrochanteric muscles result in varus deformation of the medial fragment in vivo [19]. On the other hand, the forces and moments acting at the hip joint lead to leg shortening through vertical femoral head displacement. The joint capsule adds a rotational component to fracture displacement. With less fracture displacement over a predefined number of test cycles and more cycles until fulfilment of predefined fracture displacement criteria, the artificially created fractures seem to be biomechanically more stable over osteotomies. Increased interdigitating within the rough trabecular fracture surface results in higher interlocking strength and might be the reason for better biomechanical stability with regard to fracture displacement. In turn, the higher biomechanical stability might result in less data scattering, which was apparent in the fractured group with its pooled SDs being significantly smaller compared with the osteotomized group. Consequently, more predictive results may be achieved with fractured versus osteotomized specimens, although it might seem counterintuitive at first sight due to a higher standardization in osteotomies.

This study has some limitations, inherent to those for all cadaveric investigations with limited number of used specimens, being incapable to completely simulate in vivo situations with surrounding soft tissue following a bone fracture. Moreover, the authors clearly state that the artificially created fractures did not obey the physical laws of clinical fracture pathomechanics – due to the lacking realization of standardization. Further, the current study focused on creation of only one pattern representing a simple stable pertrochanteric fracture type AO/OTA 31-A1. In addition, the used simplified biomechanical test model did not consider all muscle forces and moments acting on the femur. Regarding the observed catastrophic failure modes, all specimens failed in a combination of varus deformation, leg shortening and implant bending at the region where the lag screw extended from the barrel. Although varus deformation and leg shortening are commonly seen as in vivo failure modes following pertrochanteric fracture fixation, implant bending occurs rather seldom. Plastic deformation of the lag screw in the region where it extended from the barrel was reported in previous biomechanical work concluding that this mode of failure was rather related to the screw placement and bone quality than to the loading methodology [25]. Therefore, the observed implant failure without accompanying bone failure (e.g. cut-out) might be explained with the relatively high BMD of the specimens used in the current study. Moreover, the bone microarchitecture in general and specifically within the proximal femur is highly complex and diverse for each specific individual [26, 27]. Therefore, it was not always possible to generate exactly the same fracture patterns with special regard to the extension of the lesser trochanter. However, the involvement of this anatomical structure did not significantly deteriorate the stability of the fractured bone during biomechanical testing. This fact supports the statement that the integrity of the lateral wall of the proximal femur outweighs an intact lesser trochanter as stated in the definition of a stable pertrochanteric fracture [9].

## Conclusions

Standardized stable pertrochanteric femur fracture creation demonstrates a trend towards different biomechanical behavior as compared with fracture simulation via osteotomization and following DHS fixation. From a biomechanical perspective, by resulting in more consistent outcomes under dynamic loading, standardized artificial stable pertrochanteric femur fracture creation may be more suitable for orthopaedic implant testing compared to osteotomizing the bone.

## Data Availability

The datasets used and/or analysed during the current study are available from the corresponding author on reasonable request.
